# Analysis and prediction of addiction among virtual reality users

**DOI:** 10.1371/journal.pone.0318117

**Published:** 2025-03-12

**Authors:** Jing He, Shuman Yu, Jingzhao Zhang

**Affiliations:** 1 Key Laboratory of Philosophy and Social Science of Anhui Province on Adolescent Mental Health and Crisis Intelligence Intervention, Hefei Normal University, Hefei, China; 2 Guangxi Key Lab of Multi-source Information Mining & Security, Guangxi Normal University, Guilin, China; 3 Institute for Advanced Studies in Humanities and Social Science, Beihang University, Beijing, China; 4 School of Science, Engineering, and Medicine, The University of Warwick, Coventry, United Kingdom; Ladoke Akintola University of Technology Teaching Hospital: LAUTECH Teaching Hospital, NIGERIA

## Abstract

**Objective:**

To understand the addiction situation and influencing factors of virtual reality users, and provide reference basis for timely and effective prevention and intervention of user addiction.

**Methods:**

Based on a questionnaire survey, univariate analysis, multivariate analysis, and model prediction were conducted on the data of 1164 participants in VR related Facebook groups and Reddit subedits.

**Results:**

The single factor analysis results show that the user’s own attributes, usage duration, perception level, and application types of virtual reality devices can significantly affect the degree of addiction; The results of multivariate analysis showed that the age of users, the number of days used per week, the number of hours used per day, and the perceived level of the device can significantly affect the probability of addiction. In addition, this study used decision tree algorithm to predict adolescent virtual reality device addiction, with a prediction accuracy of 0.957.

**Conclusion:**

The addiction of virtual reality users is related to multiple factors such as gender, age, usage time, application type, and perception level. When developing VR applications and content, consideration should be given to balancing user immersion and healthy use, and reasonable control of usage time is also an effective means to prevent VR addiction.

## 1. Introduction

Virtual reality (VR is short for virtual reality) is a technology that can create an immersive experience in a computer simulation environment, using special equipment and software to allow users to enter a completely virtual world and carry out various activities and interactions in it. In recent years, with the continuous development and popularization of virtual reality technology, it has become a popular way of entertainment and learning, and has been widely used in many fields, such as games, education, medical treatment, architecture and so on [1,2]. But at the same time, some users may have virtual reality addiction problems and affect their normal life. Based on linear regression analysis and decision tree algorithm, this study explores the possible influencing factors and risk trends of virtual reality technology addiction, aiming to provide scientific basis and reference for the prevention and treatment of virtual reality addiction, and protect the physical and mental health of virtual reality users [3].

## 2. Research objects and variables’ settings

### 2.1. Research objects

This research uses publicly available dataset published by Miguel Barreda-Ángeles and Tilo Hartmann (2022) on *Frontiers in Virtual Reality* for analysis [4]. The data set is entitled *Addiction to VR applications*.1,164 participants (approximately 80% of whom were from the United States, Canada, and the United Kingdom) were recruited through posts from VR-related Facebook groups and Reddit subforums over a period from February 2022 to March 2022 [5]. The methods of questionnaire survey and data filtering were used for data collection and processing, and a dataset containing 754 groups of samples was finally collected. For more information about this dataset, refer to the online repository Open Science Framework (https://osf.io/u2pb3/). During data processing, the rows with the “other” field of 1 were deleted, and the data with gender (male, female) information was screened out, and 702 valid samples were finally obtained. 70% of the samples (491 samples) were randomly selected as the training set for training logistic regression and decision tree models. The remaining 30% (211 samples) was used as a test set to evaluate the predictive effect of the decision tree model [6,7].

### 2.2. Variables and research questions

#### 2.2.1. Object variable.

The evaluation of addiction degree is the target variable of the prediction study [8].

GAS7.1 to GAS7.7: Refers to the seven questions in the questionnaire used to assess virtual reality addiction. The GAS, which stands for Game Addiction Scale, is a commonly used tool to measure game addiction. In this study, GAS was adapted according to the characteristics of virtual reality technology, and 7 questions were formed to evaluate virtual reality addiction.

GAS7_sum: Defining a virtual reality addiction score summation variable. By adding up the scores of each sample user on seven different aspects(GAS7.1- GAS7.7). Therefore, GAS_sum can be used as a comprehensive indicator to reflect the degree of virtual reality addiction of participants.

Polythetic: Multicategory refers to a method of combining a number of different characteristics or criteria to define a concept or classification. In this study, this variable indicates whether the user is addicted due to multiple factors (1 means yes, 0 means no).

#### 2.2.2. Characteristic variables.

The degree of addiction of virtual reality users is taken as the target variable, and the definitions of relevant variables are shown in Table 1. The following factors are considered by academics to be closely related to affecting virtual reality addiction and will be argued in detail in this paper [9–11]: User attributes, usage duration, application category, spatial perception level and embodied perception level are selected as characteristic variables, mainly based on the following considerations: ① User attributes include gender, age, education level, etc. These attributes reflect the user’s cognition and demand level for virtual reality, and affect the risk of addiction. ② The length of use of virtual reality reflects the degree of interaction between an individual and the technology, and the user’s use time affects the risk of addiction. ③ The category of virtual reality application reflects its attractiveness and addiction potential, and the user’s choice and preference and the type of application affect the risk of addiction. ④ Spatial perception level refers to an individual’s ability to perceive and understand space and location in the virtual reality environment. Embodied perception level refers to the degree to which individuals perceive their own existence and sense of body in virtual reality. High or low levels of both affect the risk of addiction [12,13].

**Table 1:** Description of variables.

Variables	Classification	Necessary Description
User Attribute	Age	The sample in the dataset was between 18 and 86 years old
	Gender	There are four gender options in the data set: male, female, no gender/ the third gender
	Nation	A total of 52 countries appear in the dataset
Using Time	Days of use per week	The number of days the user uses the virtual reality device per week
	Hours of use per day	The number of hours the user uses a virtual reality device per day
	Hours of use per week	The number of hours the user uses a virtual reality device per week
Application Category	game	The number of apps in the “games” category
	socializing	The number of apps in the “social” category
	else	The number of apps in “Other” category
The level of Spatial perception [8]	Spatial perception	Describe the spatial perception level of sample users, represented by numbers 0-4. The larger the number, the higher the perception level.
The level of self-perception [8]	self-perception	Describe the self-perception level of sample users, represented by numbers 0-4. The larger the number, the higher the perception level.

#### 2.2.3. Research questions.

Compared with other media technologies, the characteristics of VR applications that motivate users to have a strong sense of interaction and make them gain emotions have intensified users’ concerns about VR addiction. VR addiction among adolescents has a higher risk and greater impact. Understanding youth use of VR devices and related factors is important for the development of appropriate health and safety policies. Therefore, based on the above research variables, the following research questions are proposed:

Q1：What factors affect user addiction?
Q2：How is addiction among adolescents?
Q3：Predict addiction to virtual devices.

## 3. Result

### 3.1. Single factor analysis of virtual reality equipment use addiction

#### 3.1.1. The influence of user’s own attributes on the degree of addiction.

**Fig 1:**
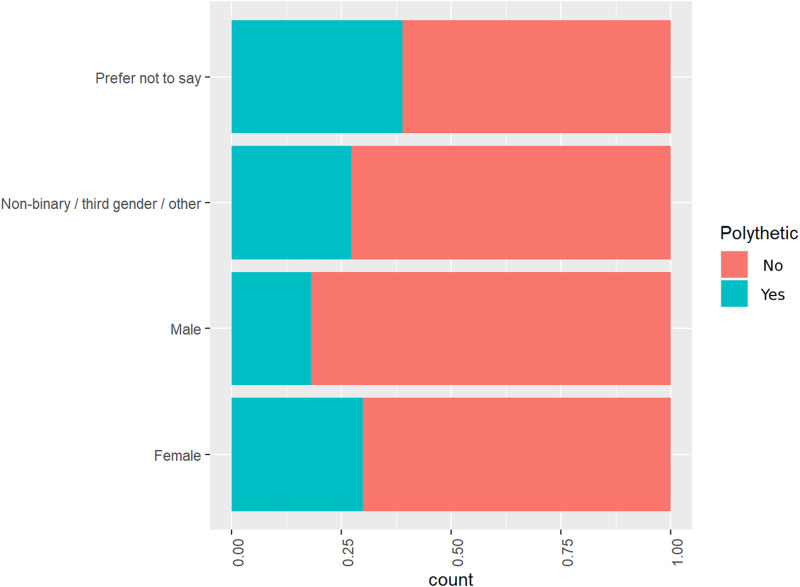
Gender distribution of addicted users.

**Fig 2:**
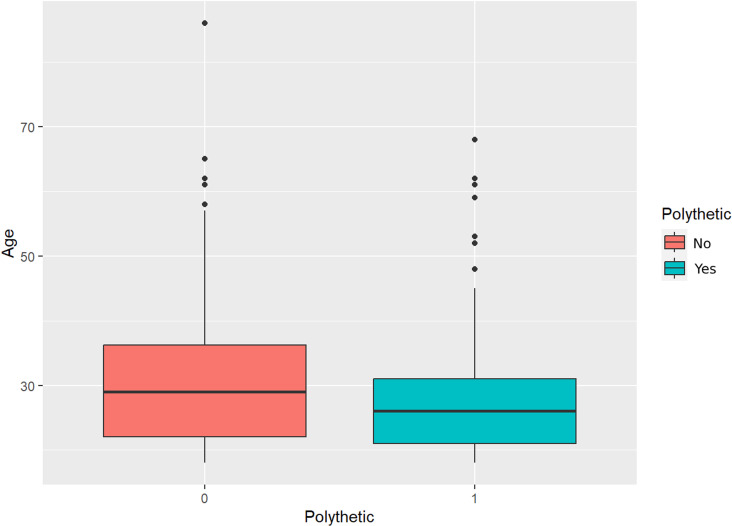
Age distribution of addicted users.

In the training set, the variables of age, sex and country were fitted into a binary logistic regression model. The results show that both the numerical variable age and the binary variable gender-male significantly affect whether users are addicted or not. But the country variable has no significant effect on user addiction. In the box plots in Figs 1 and 2, green indicates that the user is showing symptoms of addiction, and red indicates that the user is not showing symptoms of addiction.

From Fig 1, it can be seen that for users of different genders, The addiction rate of VR devices varies, with gender sensitive users having the highest VR addiction rate and males having the lowest VR addiction rate, specifically 18%; The regression analysis results indicate that the older the user is, the lower the risk of addiction.

From the comparison bar chart 2, it can be seen that users with obvious VR addiction are younger in age; The regression analysis results indicate that men are less likely to be addicted than women.

#### 3.1.2. The effect of the length of time users use VR devices on the degree of addiction.

A binary logistic regression model was fitted to the variables of days of use per week, hours of use per day and hours of use per week. The results of regression analysis showed that the number of days per week and the number of hours per day significantly affected whether users were addicted or not. The longer the number of days per week and the longer the daily use of VR devices, the greater the risk of addiction.

#### 3.1.3. The influence of user perception level on the degree of addiction.

The analysis shows that users’ spatial perception level and physical awareness level can significantly affect users’ degree of VR device addiction. The regression analysis results show that the higher the user’s spatial perception level score and the higher the user’s physical awareness level score, the greater the risk of addiction.

#### 3.1.4. Effects of virtual reality technology application types on user addiction.

**1. Using time of different types of application**

Discussing whether the use time of game apps (hereinafter referred to as “game timing”), social apps (hereinafter referred to as “social timing”) and other apps (hereinafter referred to as “other timing”) [14,15] has statistical significance in terms of users’ age, gender, country, spatial perception level, and physical awareness level (P <  0.001 or P < 0.05) as shown in Table 2.

**Table 2:** The impact of each variable on the usage time of different apps.

Independent variable	Regressive analysis	Gaming category	Social category	Other categories
Age	The value of β	−0.02934152	−0.02309335	−0.02898895
	Standard error	0.00799359	0.01663704	0.04234776
	The value of t	−3.6706306	−1.3880687	−0.6845451
	The value of p	0.000271044	0.1667331	0.49722116
Gender-Male	The value of β	−0.23362035	−0.14415696	−0.44939837
	Standard error	0.26745256	0.41678162	1.4048189
	The value of t	−0.873502	−0.3458813	−0.3198977
	The value of p	0.3828564	0.7298126	0.75055998
Nation	The value of β	0.13124356	0.36492907	0.93091205
	Standard error	0.15438469	0.3449695	0.7973771
	The value of t	0.8501074	1.0578589	1.1674677
	The va;ue of p	0.3957185	0.2914558	0.24930937
The level of Spatial perception	The value of β	0.12751476	−0.12330798	−0.35857387
	Standard error	0.09043984	0.19487269	0.37752014
	The value of t	1.4099402	−0.6327617	−0.9498139
	The value of p	0.1592497	0.5276461	0.34739499
The level of self-perception	The value of β	0.08405153	0.26193444	0.6258002
	Standard error	0.08032334	0.16749059	0.29079308
	The value of t	1.0464148	1.5638755	2.1520464
	The value of p	0.2959325	0.1195021	0.0369202

Perform linear regression analysis using game time as the target variable. The results showed that at a significance level of α=0.05, only age had a significant impact on the daily usage time of gaming app users (P value < 0.001), and the two were negatively correlated.

Perform linear regression analysis using social reporting hours as the target variable. The results showed that at a significance level of α=0.05, the independent variable had no significant impact on social reporting hours. The fitting effect of this model is poor, with a multiple R-squared value of 0.02847 and an adjusted R-squared value of 0.003041, The p-value of the F-statistic is 0.3514.

Perform linear regression analysis with other reported hours as the target variable. The results showed that at a significance level of α=0.05, only the level of embodied awareness had a significant impact on the average daily usage time of other types of app users, and the two were positively correlated.

**2. Addiction to different types of applications**

Through a Welch’s t-test comparing addiction scores for different types of apps, it was found that at a significance level of α=0.05, the level of addiction among social app users (with an average GAS-7 total score of 14.97) was significantly higher than that among gaming app users (with an average GAS-7 total score of 12.63); The difference in addiction level between social app users (average GAS-7 total score of 14.97) and other app users (average GAS-7 total score of 13.46) is insignificant.

The addiction caused by users using different types of apps is represented by the total GAS7 score (GAS7_sum) value. The Welch’s Two Sample t-test results of the total GAS7 scores between different genders showed that at a significance level of α=0.05, the addiction detection rate of gaming apps was 14.5% (66/455), with an average addiction score of 12.63077. There was no significant difference in average scores between male and female users; The addiction detection rate of social apps is 31% (61/197), with an average addiction score of 14.96954. The average score of female users is significantly higher than that of male users; The addiction detection rate of other types of apps is 20% (10/50), with an average addiction score of 13.46. The difference in average scores between male and female users is also not significant. Overall, there are significant differences in user addiction among different types of applications, and social apps are more likely to lead to addiction among female users.

### 3.2. Multiple factors analysis of the use of virtual reality devices

A multivariate binary logistic regression model was established on the training set using variables that were significant in single-factor analysis. Considering multicollinearity, the variable of weekly usage hours was removed from the study. (Since weekly usage is a direct aggregate of daily usage, both variables convey highly overlapping information. Daily usage time was chosen for its finer granularity, providing more precise insights into user behavior patterns and offering greater explanatory power in the analysis) [16,17].

From the results, it can be seen that age, number of days used per week, number of hours used per day, and embodied perception level have a significant impact. From the regression coefficients, it can be seen that for age: the corresponding OR value is 0.957, with a 95% confidence interval of [0.929864241, 0.9831972], indicating that when other variables are fixed, for every 1 year increase in age, P (addicted)/p (not addicted) is 0.957 times the original. The same conclusion is as follows. For the number of days used per week: the corresponding OR value is 1.53794119, with a 95% confidence interval of [1.335912044, 1.7841552]. For daily usage hours: the corresponding OR value is 1.15457077, with a 95% confidence interval of [1.021295826, 1.3084566]. For embodied perception level: the corresponding OR value is 1.46819248, with a 95% confidence interval of [1.139444109, 1.9027451].

### 3.3. Analysis of factors related to adolescent addiction to virtual reality devices

Perform multiple linear regression analysis on adolescent data in the age range of 10 to 19 years old.

#### 3.3.1. Multiple linear regression analysis using time related factors.

Multiple linear regression analysis was conducted using the number of hours of using virtual reality devices per week as the dependent variable, and seven items of adolescent age, gender, nationality, spatial perception level, embodied awareness level, and game addiction scale as independent variables. The results are shown in Table 3. Specifically, based on the average amount of time spent using virtual reality devices per week, adolescents from Europe (9.68) and North America (13.99) have longer usage time compared to adolescents from Australia (4.8); When the spatial perception level score is ≥  2 and the embodied perception level score is less than or equal to 3, it will significantly reduce the time adolescents spend using virtual reality devices (12.23).

**Table 3:** The impact of each variable on adolescent VR addiction.

Independent variable (adolescent)	Virtual reality device usage time			Addiction to virtual reality devices		
The value of β	Standard error	The value of t	The value of β	Standard error	The value of t
Age	1.0620	2.5269	0.4200	−1.9300E-16	3.5910E-16	−5.3800E-01
Gender	−0.2638	3.6367	−0.0730	−1.8910E-15	5.1680E-16	−3.6590E + 00
The level of Spatial perception	−1.6410	1.4920	−1.1000	−5.3690E-16	2.1200E-16	−2.5320E + 00
The level of self-perception	−0.7432	1.0176	−0.7300	−2.6360E-17	1.4460E-16	−1.8200E-01
Nation	1.3614	2.4407	0.5580	8.5410E-16	3.4690E-16	2.4620E + 00
GAS7.1	2.4040	1.4775	1.6270	1.0000E + 00	2.1000E-16	4.7620E + 15
GAS7.2	4.6221	1.5761	2.9320	1.0000E + 00	2.2400E-16	4.4640E + 15
GAS7.3	−0.0644	0.8686	−0.0740	1.0000E + 00	1.2340E-16	8.1010E + 15
GAS7.4	1.7936	2.0340	0.8820	1.0000E + 00	2.8910E-16	3.4590E + 15
GAS7.5	−0.2240	1.4063	−0.1590	1.0000E + 00	1.9990E-16	5.0030E + 15
GAS7.6	5.6765	3.5119	1.6160	1.0000E + 00	4.9910E-16	2.0040E + 15
GAS7.7	−1.0985	1.1347	−0.9680	1.0000E + 00	1.6130E-16	6.2010E + 15

Regression analysis shows that only GAS7.2 (a sub factor of GAS7) has a significant impact on the usage time of adolescent devices. The higher the value of this variable filled in by adolescent users, the longer their VR device usage time will be. In behavioral psychology, The GAS7.2 indicator represents the “stimulus” dimension, which refers to the degree to which users seek novelty and stimulation in virtual reality. This indicator is related to the personality traits and living environment of adolescents, and its impact on the use of virtual devices by adolescents mainly includes: the higher the GAS7.2 indicator, the more likely adolescents are to develop dependence and addiction to virtual devices, leading to psychological health problems; The more likely it is to overlook responsibilities and obligations in real life, the more difficult it is to achieve growth and development; The more difficult it is to adapt to setbacks and difficulties in real life, the lack of coping ability and resilience [18,19].

#### 3.3.2. Analysis of factors related to addiction scores.

Use linear regression analysis to explore the various factors that affect the total addiction score (GAS7_sum) in adolescent samples. After data preprocessing, 57 sample data were screened. In the multiple regression model, seven items of adolescent age, gender, nationality, spatial perception level, embodied awareness level, and game addiction scale were used as independent variables to explore their impact on the dependent variable GAS7 total score (GAS7_sum). The results are shown in Table 3. Specifically, using the average value as the evaluation criterion, compared to adolescent boys, adolescent girls have a more severe addiction to virtual reality devices; Compared to Australia (19) and the Americas (15.21), European teenagers have a lower level of addiction (13.85); Adolescents(16) with higher levels of spatial perception (3-4) have a higher level of addiction than adolescents(13.73) with higher levels of spatial perception (0-2); Teenagers (17.36) with higher levels of embodied awareness (3-4) have higher levels of addiction than those(14.39) with lower levels of embodied awareness (0-2) [20].

The regression results indicate that, with α=0.05 as the significance level, the coefficient p-values of gender, country, and spatial perception level are < 0.05, all of which have significant statistical significance. Gender, country, and spatial perception levels have a significant impact on the total score of GAS7. The coefficient p-value of age and embodied perception level is greater than 0.05, indicating that age and embodied perception level have no significant impact on the total score of GAS7. GAS7.1 to GAS7.7 have a significant impact on the total score of GAS7, The p-value is less than 0.001, and the t-values are all very large. Overall, gender, country, spatial perception level, and the seven sub factors of GAS7 all have a significant impact on adolescent virtual reality device addiction [21].

### 3.4. Prediction of addiction probability for virtual reality devices

A multivariate binary logistic regression model was established using significant variables in univariate analysis on the training set (491 samples in total). The test set consisted of 211 samples, with 170 non addicted samples. Logistic regression correctly predicted 166, but incorrectly predicted 4 non addicted users as addicted. There are 41 addictive samples in the test set, and logistic regression correctly predicted 41 of them, incorrectly predicting 2 addictive users as non-addictive. It can be seen that the prediction accuracy of logistic regression on the test set is 0.972.

**Fig 3:**
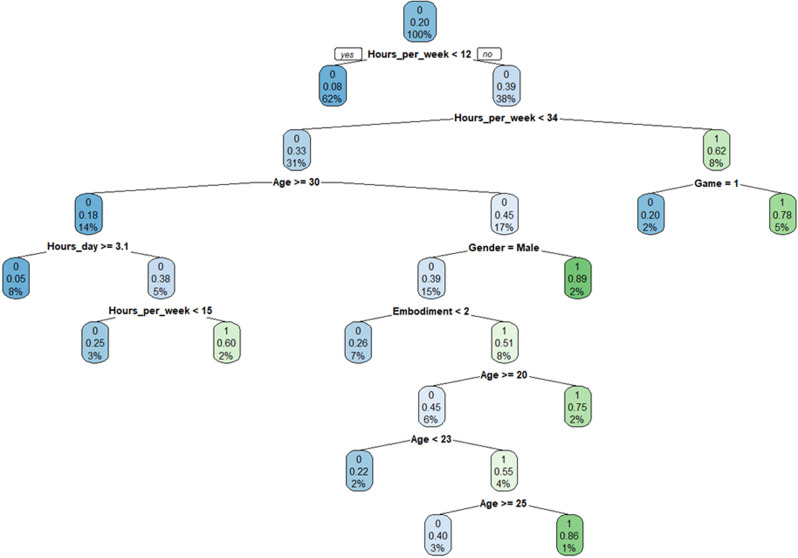
The model of decision tree for predicting addiction to virtual reality devices.

This study uses decision tree algorithm to predict the addiction of adolescents to virtual reality devices. The decision tree model is generated as shown in Fig 3, where features include age, gender, daily usage duration, weekly usage duration, whether the application used is of a game type, and embodied awareness level. The target variable is a multi-category, with values set to 0 or 1, where 0 represents a non-addictive state and 1 represents an addictive state. This decision tree algorithm can predict the likelihood of adolescents becoming addicted to virtual reality devices through a series of variables, thus facilitating the provision of relevant addiction prevention strategies. The blue color of the node indicates a low probability of addiction, the green color indicates a high probability of addiction, and the depth represents the size of the probability.

The highest probability of addiction predicted in this decision tree occurs under the conditions of 12 ≤  weekly usage hours < 34&age < 30&gender ≠  male, that is, 12 ≤  weekly usage hours < 34&age < 30&gender=female. Users who meet this condition have a high probability of becoming addicted, with this type of user accounting for 2% of all users in the training set.

Test and evaluate the decision tree model. This study uses the decision tree model mentioned above to predict whether users in the test set are addicted, and evaluates the predictive performance of the model. When a new sample is given, based on the value of the input variable, the decision tree model will divide the sample into a leaf node according to the established decision rules, and then classify and predict according to the category represented by the leaf node. The decision tree correctly predicted 166 out of 170 non addicted samples in the test set, but incorrectly predicted 4 non addicted users as addicted. There are 41 addictive samples in the test set, and the decision tree correctly predicted 36 of them, incorrectly predicting 5 addictive users as non addictive. It can be seen that the prediction accuracy of this decision tree on the test set is 0.957.

## 4. Discussion

This study reveals that VR addiction is influenced by a combination of demographic, behavioral, and perceptual factors. In contrast to previous studies, this research provides a multifaceted understanding of addiction by integrating user attributes, behavior, and perceptual dimensions. It offers a refined analytical approach and a broader perspective on managing VR addiction.

Single-factor analysis highlights significant associations between addiction and variables such as younger age, female gender, extended usage durations, and higher spatial and embodied perception levels. Social apps are found to have a greater addictive potential than other application types.

Through multi-factor analysis, the combined effects of these variables are confirmed, with younger users, longer usage hours, and higher embodied perception levels identified as key contributors to addiction risk. Adolescents, in particular, are shown to have higher susceptibility, driven by novelty-seeking tendencies and perceptual engagement. Regional differences also play a role, with adolescents in North America and Europe displaying longer usage durations compared to their Australian counterparts.

The decision tree model used for prediction achieves an accuracy of 95.7%, effectively identifying high-risk groups, such as younger female users with moderate weekly usage durations. These findings offer valuable insights for targeted interventions and emphasize the need for balanced VR design to mitigate addiction risks.

Although the accuracy of the decision tree model indicated that the model has strong predictive ability for new samples and high accuracy, it should be noted that there are still some limitations in this study. Firstly, the dataset and methods used in the study may have certain biases and limitations. Secondly, due to the rapid development of virtual reality technology, future research needs to focus on the addiction risks of emerging VR devices and applications, and further explore other potential influencing factors [22]. In addition, more long-term tracking studies are needed to gain a deeper understanding of the development patterns and impact mechanisms of VR addiction.

## 5. Conclusion and suggestion

### 5.1. General conclusions for single factor

#### 5.1.1. Age factor.

There is a negative correlation between age and VR addiction: as individuals grow older, their risk of becoming addicted to VR devices decreases. This finding aligns with common perceptions and is supported by statistical analysis. Several physical and mental factors may contribute to this trend. Older individuals often experience discomfort, such as motion sickness or difficulty using VR headsets [23], and may exhibit reduced curiosity and interest in exploring new technologies. Additionally, increased responsibility and maturity with age could play a role.

Generational differences also influence VR addiction, as younger and older generations differ in cultural norms, socialization habits, and technological literacy. However, the age-related factors may diminish over time as future generations become accustomed to advanced technologies like VR from a young age.

Despite these changes, the author believes that the inverse correlation between age and the risk of VR addiction will persist, albeit in increasingly complex ways. Therefore, VR developers should tailor experiences to different generations and age groups. For younger users, strategies to enhance self-control and manage screen time should be emphasized. For older users, VR experiences should promote balance, offering shorter, purpose-driven sessions for activities such as relaxation, fitness, or learning.

#### 5.1.2. Gender factor.

In terms of gender, male users have a lower likelihood of VR addiction compared to female users, and the risk of male addiction is only about 0.428 times that of female users.

In terms of the social roles, women are often associated with multiple responsibilities, which may lead them to seek out VR as a means of stress relief, socializing, or self-expression. Male users may not face the same level of emotional or social pressures to the same extent, making VR a more casual, less immersive experience for them.

As a result, female users tend to possess stronger emotional engagement with immersive experiences in order to seek emotional connection, social interaction, and escapism from VR, making them more susceptible to prolonged and excessive use. This contrasts with male users, who might be more inclined toward VR experiences that focus on competition or action, which may not foster the same level of emotional attachment or sustained engagement.

A number of studies have pointed out that women may also be more sensitive to the sensory stimulation provided by VR [24], which could contribute to stronger emotional and psychological attachment to the experience, leading to prolonged use.

In light of the complexity of gender-related issues, it is quite limiting to speculate on why women are more likely to be addicted to VR through data analysis and gender macro-differences alone.VR developers should evaluate specific psychological and contextual factors of the user group based on characteristics such as different content preferences or different interaction modes of the project.

#### 5.1.3. Usage behavior factor.

There is a positive correlation between usage time and VR addiction. The longer the number of days a user uses per week and the duration of daily use, the higher the risk of addiction to VR devices. This result is consistent with the findings of other studies in the field of Internet and mobile phone addiction, that is, there is a close relationship between overuse and addiction. Reasonable control of usage time and avoidance of excessive addiction to VR experience are crucial for preventing and managing VR addiction.

#### 5.1.4. Perceptual level factor.

perceptual level also has a significant impact on VR addiction. There is a positive correlation between an individual’s spatial perception level and embodied awareness level and VR addiction. Individuals with higher levels of spatial perception and embodied awareness are more likely to become addicted to VR devices. This may be because these individuals are more likely to immerse themselves in virtual environments, experiencing a stronger sense of presence and identity. Therefore, when developing VR applications and content, consideration should be given to balancing user immersion and healthy use.

### 5.2. Specific conclusion for adolescent addiction

Through further exploration of factors related to adolescent addiction to virtual reality devices, the results showed that adolescents in Europe and North America have longer usage times compared to adolescents in Australia. Meanwhile, when the spatial perception level score is ≥  2 and the embodied perception level score is less than or equal to 3, it will significantly reduce the usage time of adolescents. Regression analysis also showed that only one sub factor in the Game Addiction Scale (GAS7.2) had a significant impact on the duration of adolescent device use, that is, the higher the value of this indicator, the longer the duration of adolescent virtual reality device use. This indicator represents the degree to which adolescents seek novelty and stimulation in virtual reality, which is related to their personality traits and living environment. In the analysis of factors related to addiction scores, linear regression analysis was used to explore the factors that affect the total addiction score. The results showed that gender, country, and spatial perception level had a significant impact on the total score of addiction, while age and embodied perception level had no significant impact on the total score of addiction. The sub factors of the Game Addiction Scale have a significant impact on the total score of addiction [25].
